# Characterization of the position of the extraocular muscles and orbit in acquired esotropia both at distance and near using orbital magnetic resonance imaging

**DOI:** 10.1371/journal.pone.0248497

**Published:** 2021-03-12

**Authors:** Manami Kawai, Toshiaki Goseki, Hitoshi Ishikawa, Sonoko Tatsui, Hongyang Li, Ryutaro Ukisu, Nobuyuki Shoji

**Affiliations:** 1 Department of Ophthalmology, Kitasato University School of Medicine, Sagamihara, Kanagawa, Japan; 2 Department of Ophthalmology, International University of Health and Welfare Atami Hospital, Atami, Shizuoka, Japan; 3 Department of Ophthalmology, Beijing Friendship Hospital, Capital Medical University, Beijing, Xicheng District, China; 4 Department of Diagnostic Radiology, Kitasato University School of Medicine, Sagamihara, Kanagawa, Japan; Faculty of Medicine, Cairo University, EGYPT

## Abstract

**Purpose:**

Age-related distance esotropia (ARDE) involves acquired esotropia at distance and phoria at near. However, distance-independent esotropia (DIE) exists esotropia both at distance and near. Thus, we examined the orbital magnetic resonance imaging (MRI) findings for DIE to assess differences in its characteristics.

**Methods:**

This study was a retrospective case-control study. We evaluated the efficacy of the standard coronal MRI in patients with acquired esotropia and control patients with optic neuritis. Cases with strabismus in the control group were excluded. DIE was defined as having esotropia both at distance and near, and an angle of more than 10 prism diopters at near. The condition of the lateral rectus-superior rectus band, position of rectus muscles, and the volume ratio of the globe to the whole orbit (G/WO) were examined.

**Results:**

The DIE group consisted of 12 eyes of 6 patients (77.3±7.7 years); ARDE group, 38 eyes of 19 patients (73.1±6.8 years); and control group, 34 eyes of 17 patients (70.9±4.3 years). The ratio of abnormality of the lateral rectus-superior rectus bands was higher in the DIE and ARDE groups than in the control group (*p*<0.01). The vertical angle of the lateral rectus deviated downwards in the control (-7.5±5.1°), ARDE (-12.2±9.1°), and DIE groups (-18.8±5.7°) (*p*<0.05). The tilting angle of the lateral rectus was tilted temporally in the control (-12.2±9.1°), ARDE (-20.0±8.6°) and DIE groups (-28.6±5.4°) (*p*<0.01). G/WO was higher in the DIE (0.28±0.01) and ARDE groups (0.27±0.02) compared to the control (0.25±0.03) group (*p*<0.01).

**Conclusion:**

In comparison with the ARDE and control groups, the DIE group presented with abnormalities of the lateral rectus-superior rectus band, malposition of the lateral rectus, and differences in the G/WO. The DIE group showed a more severe form of ARDE.

## Introduction

Age-related distance esotropia (ARDE) is an age-related divergence paralysis-like condition that presents with esotropia at distance and phoria at near [1,2]. Rutar and Demer [3] and Chaudhuri and Demer [4] used orbital magnetic resonance imaging (MRI) to show that elderly patients with ARDE had abnormalities of the orbital connective tissue and malposition of the extraocular muscles, especially the lateral rectus (LR) muscle. This strabismus caused by age-related changes in the orbital connective tissue was categorized as sagging eye syndrome (SES), which is further distinguished into ARDE and cyclo-vertical strabismus (CVS). Other conditions occurring in addition to SES, an acquired strabismus that is associated with changes in the degeneration of the connective tissue and malposition of the extraocular muscles, include heavy eye syndrome, in which the posterior part of the globe is dislocated out of the muscle cone from between the superior rectus (SR) and the LR due to a high axial length [5], high myopic strabismus without dislocation of the posterior part of the globe [6,7], and knobby eye syndrome with irregular staphyloma [8]. These conditions can be attributed to the mechanical stress on the pulley of the orbital connective tissue due to the long axial length [9,10] and the orbit and the axial length mismatch [6,11].

In clinical practice, ARDE is a common cause of elderly binocular diplopia [12]. However, some cases may show acquired esotropia at near as well as at distance [13,14]. On exclusion of neurogenic factors, neuromuscular junction–related conditions, myogenic diseases, and axial myopia, the details of the state of the orbital connective tissue and the location of the extraocular muscles on orbital MRI scans in such cases have not been elucidated. This study aimed to compare the orbital MRI findings in cases of distance-independent esotropia (DIE) with cases of ARDE and elderly controls.

## Methods

### Participants

This case-control study was approved by the Kitasato University Ethics Committee (17–98) and adhered to the tenets of the Declaration of Helsinki (as revised in Brazil 2013). As this was a retrospective study, patient informed consent was not provided in writing, and the opt-out method was applied to obtain consent by using a poster on our website.

This study involved a retrospective analysis of consecutive patients older than 60 years with acquired esotropia who visited the Kitasato University Hospital between April 2014 and September 2019. Records were accessed to obtain the retrospective data used in this study between November 2019 and January 2020. ARDE was diagnosed if the patient presented with acquired esotropia at distance (5 m) and phoria at near (33 cm), and 38 eyes of 19 patients (male/female = 8:11) were included. In contrast, DIE was diagnosed when the patients showed esotropia both at distance and near as well as an angle of more than 10 prism diopters (Δ) at near, and 12 eyes of 6 patients (male/female = 3:3) were included. Both had normal outward saccades. In our hospital, elderly patients with esotropia were routinely undergo head and orbital MRI scans to investigate the cause of the strabismus. Exclusion criteria was a history of strabismus or surgery, neurogenic disease, neuromuscular junction disease, myopathy, divergence paralysis caused by a cerebrovascular accident or brain tumor, orbital trauma, high myopia (a subjective refraction ≥ −6.00 D or an axial length ≥ 27.00 mm [7]), or a superotemporally displaced globe on orbital MRI scans. Additionally, coronal MRI scans that were unclear and difficult to analyze were also excluded (e.g., images from a case in which the extraocular muscle was in contact with the globe) because the position of the extraocular muscle may not be measured correctly. The eye position and deviation angle were measured using the cover-uncover test and the alternate prism cover test at distance and near. Axial length was measured from the MRI scans as the distance from the anterior corneal surface to the retinal surface along a line perpendicular to the iris plane, measured in the axial image plane closest to the globe equator [15,16].

As an age-matched control group, 34 eyes of 17 patients with optic neuritis (male/female = 5:12) were compared [17]. The mean best-corrected visual acuity was 0.06 ± 0.29 (logMAR) for the non-affected eye and 0.71 ± 0.65 (logMAR) for the affected eye. Patients with a history of strabismus or trauma were excluded from the control group. Exclusion of strabismus was confirmed by the cover test before and after the treatment of optic neuritis.

### Review of MRI scans

Orbital MRI was performed without the use of quasi-coronal MRI [17]. The patient’s head was immobilized, and their eyes were closed. MRI scans were acquired by Discovery MR750w 3T (GE Healthcare, Waukesha, WI, USA) using two-dimensional fast spin echo in a T2 sequence without fat suppression and MAGNETOM Skyra 3T (Siemens Healthineers, Erlangen, Germany) using two-dimensional turbo spin echo in a T2 sequence. The parameters of acquisition for the Discovery MR750w 3T were as follows: repetition time, 3600 ms; echo time, 102 ms; number of excitation, 2, 16 slices; slice thickness, 3 mm; slice gap, 1 mm; field of view, 160 mm; acquisition matrix, 320 × 224; reconstruction matrix, 512 × 512; acquisition time, 2 min 17 s. The parameters of acquisition for the MAGNETOM Skyra 3T were as follows: repetition time, 4600 ms; echo time, 64 ms; number of excitation, 2, 15 slices; slice thickness, 3 mm; slice gap, 1 mm; field of view, 160 mm; acquisition matrix, 320 × 240; reconstruction matrix, 640 × 480; acquisition time, 2 min 47 s.

#### Measurements of ocular pulley state and position of rectus muscles

T1- and T2-weighted MRI scans were saved as JPEG files on a Windows computer (Microsoft Corporation, Redmond, WA, USA) and measured using the ImageJ image analysis software (US National Institutes of Health, Baltimore, MD, USA).

**Fig 1:**
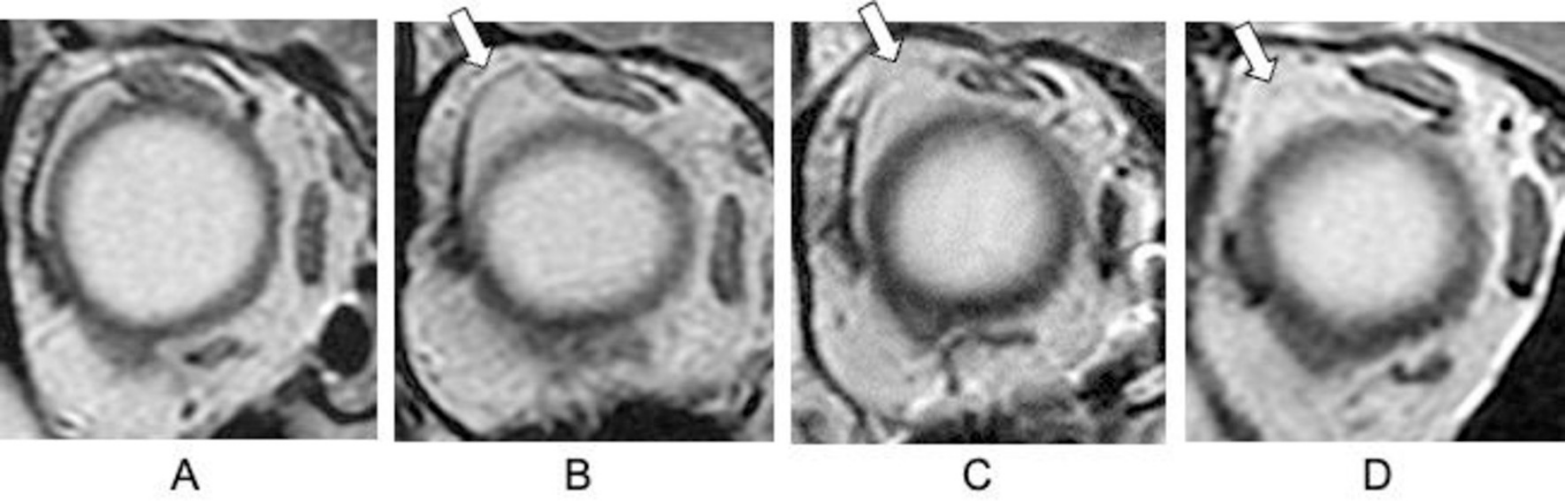
Details of the lateral rectus–superior rectus band evaluation by orbital coronal magnetic resonance imaging. (A) Control. (B) Elongation. (C) Rupture. (D) Disappearance.

The coronal plane of the orbital MRI scans was used for measurements at 3 mm to 6 mm anterior to the optic nerve attachment [18,19]. The presence of an LR-SR band was based on the following findings: continuous and not elongated, i.e., control (Fig 1A), elongation (Fig 1B), rupture (Fig 1C), and disappearance (Fig 1D) [17]. Elongation, rupture, and disappearance were considered abnormal.

**Fig 2:**
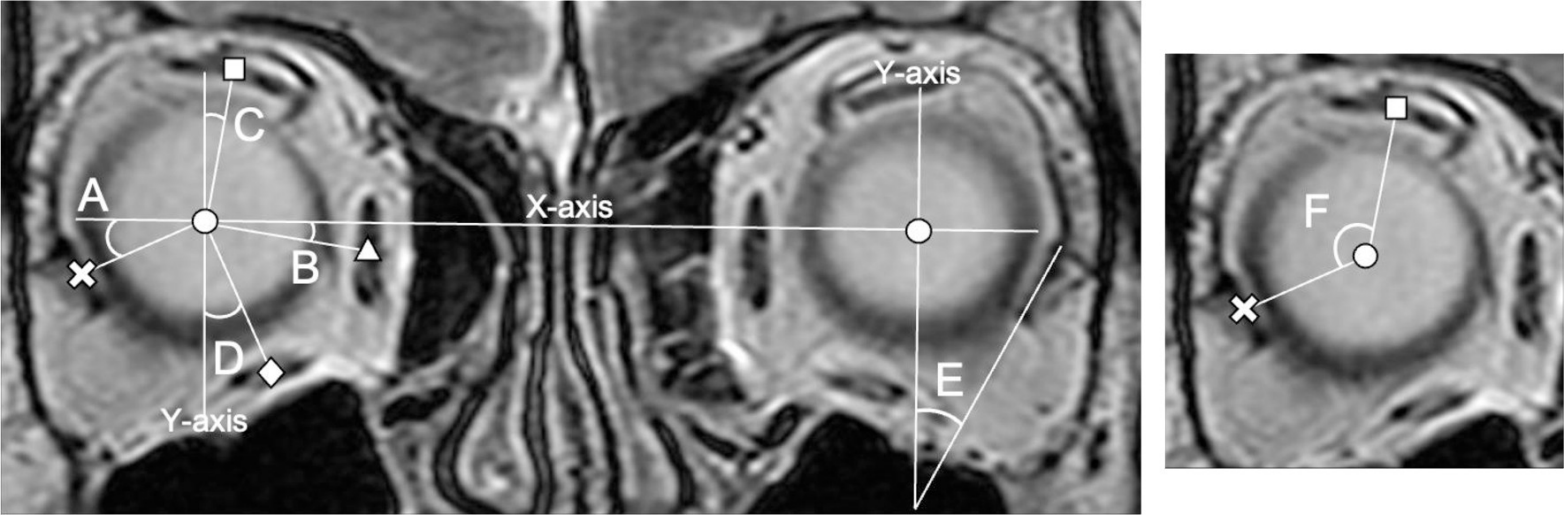
Details of the extraocular muscle malpositioning evaluation by standard coronal magnetic resonance imaging. Vertical angle of the horizontal muscle (A, B), horizontal angle of the vertical muscle (C, D), tilting angle of the lateral rectus (LR) muscle (E), and superior rectus (SR)–LR displacement angle (F). Circle (●), globe center; cross (×), LR center; triangle (▲), medial rectus center; square (■), SR center; diamond (◆), inferior rectus center.

Using ImageJ software, each rectus muscle and globe were traced using freehand selections, and their cross-sectional area center of gravity was measured using the area centroid measurement function in the pixel scale [17]. The x-axis was defined as the line connecting the centers of the two globes, and the y-axis was defined as the line passing through the center of the globe and perpendicular to the x-axis. The angle formed by the x-axis and the horizontal rectus muscles was defined as vertical angle (Fig 2A and 2B) [17]. The angle formed by the y-axis and the vertical rectus muscles was defined as the horizontal angle (Fig 2C and 2D) [20]. The angle formed by the major axis of the LR muscle and the y-axis was defined as the LR tilting angle (Fig 2E) [17]. The angle of the SR center–globe center–LR center was defined as the SR-LR displacement angle (Fig 2F) [17,21].

#### Measurements of proptosis and the globe and orbital volumes

**Fig 3:**
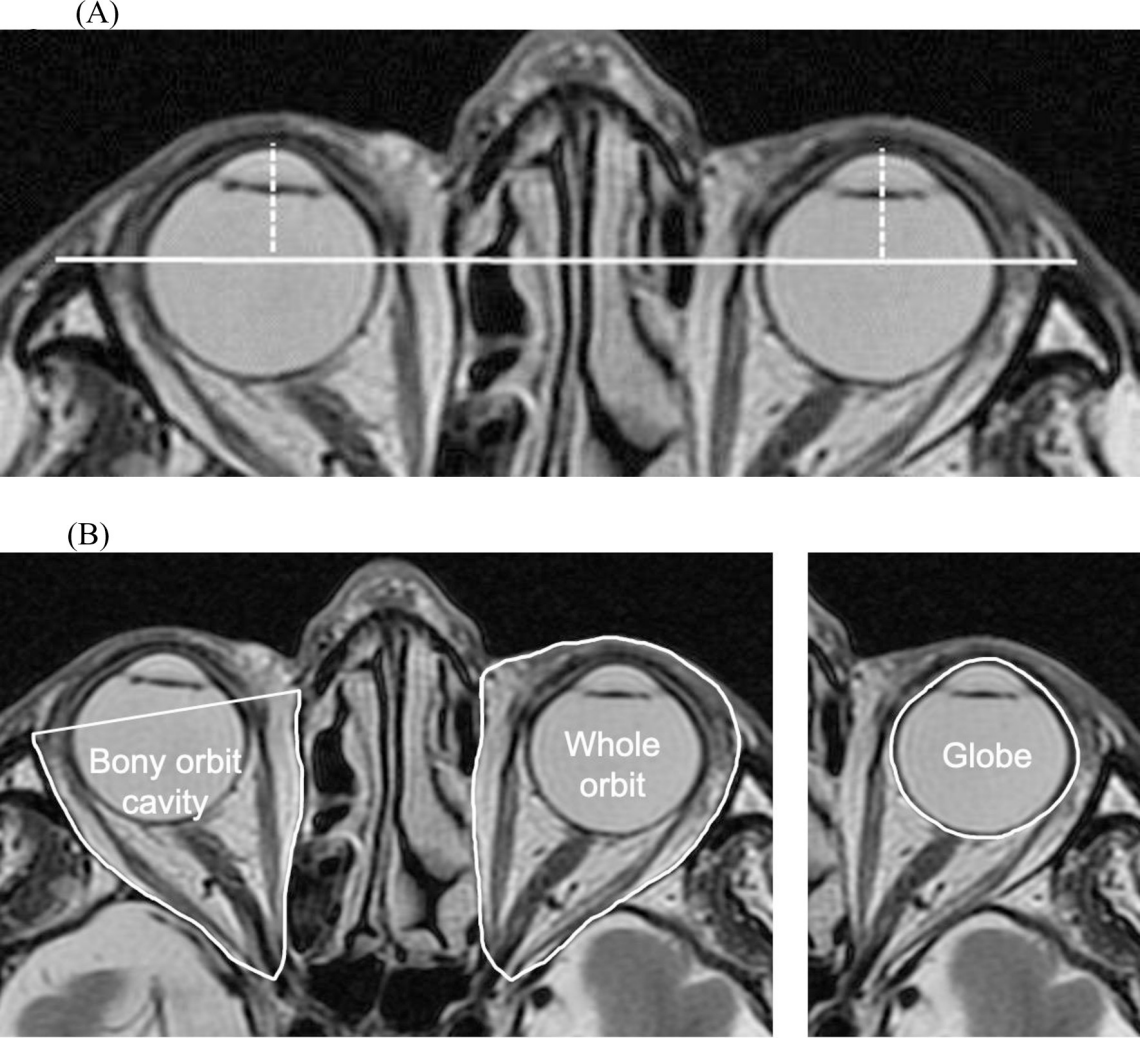
(A) Measurement of the proptosis by axial magnetic resonance imaging. A line was drawn between the bilateral frontal processes of the zygomatic bones (solid line). The proptosis value was measured as the perpendicular distance from the top of the cornea to the line (dotted line). (B) Measurement of the cross-sectional areas of the orbital tissues on T2-weighted images. Axial slice showing the cross-sectional areas of the bony orbital cavity in the right orbit and that of the globe and the whole orbit in the left orbit.

Proptosis and the areas of the globe and orbit were derived from axial and T2-weighted images using the EV Insite software (PSP Corporation, Tokyo, Japan). For assessment of proptosis, cross-sections were selected using the axial image plane closest to the globe equator. A line was drawn between the bilateral frontal processes of the zygomatic bones, and the perpendicular distance was measured from the anterior vertex of the cornea (Fig 3A) [22]. The cross-sections for the globe, whole orbit, and bony orbital cavity were traced using freehand selections, and their areas were calculated (Fig 3B) [22]. The volume of each tissue was calculated by multiplying the sum of the cross-sectional areas by the slice increment (slice thickness = 3 mm) [22,23]. There values were increased by 25% because of a 1-mm slice gap per 4 mm. The ratio of the volume of the globe to that of the bony orbital cavity (G/BO) or whole orbit volume (G/WO) was calculated. A certified orthoptist and an ophthalmologist (MK and TG), who were blinded to the clinical data, drew the regions of interest in these slices.

### Statistical analysis

All the reported values and error bars on the graphs were presented as mean ± standard deviations. One-way analysis of variance and Bonferroni post-hoc test were used to analyze cohort differences in age, axial length, angle value of extraocular muscles, proptosis, and volumes. Student’s t test and Mann-Whitney U test were used to analyze the deviation angle as well as the intergroup differences in the interval between the onset of diplopia and the visit to our hospital. Fisher’s exact test was used to analyze differences in the number of abduction limitations and abnormalities of the LR-SR band. The statistical programming language "R" (version 3.4.0; The Foundation for Statistical Computing, Vienna, Austria) was used for all statistical analyses. The statistical significance level was set at *p* < 0.05.

## Results

### Clinical characteristics

The patient’s background characteristics are shown in Table 1. There were no significant intergroup differences in age, axial length, and interval between the onset of diplopia and the visit to our hospital. The deviation angles at distance and near were greater in the DIE group than in the ARDE group (*p* < 0.01). The abduction limitations were -1 and -2 in 9 and 3 eyes, respectively, in the DIE group. One of the 38 eyes showed an abduction limitation of −1, the other 37 eyes showed no limitation in the ARDE group. There was a significant difference between the two groups (*p* < 0.01).

**Table 1:** Characteristics of patients in each group.

	DIE	ARDE	Control	*p* value
**Age (years old)**	77.3±7.7	73.1±6.8	70.9±4.3	0.09
**Axial length (mm)**	23.3±1.0	23.3±1.3	23.5±1.0	0.85
**Period until first visit (month)**	78.8±59.9	75.4 ±75.9		0.92
**Abduction limitation (number of eye)**	12	1		<0.01**
**Horizontal strabismus angle (distance) (Δ)**	+34.2±5.8	+8.5±5.0		<0.01**
**Horizontal strabismus angle (near) (Δ)**	+19.0±4.8	-1.2±5.8		<0.01**

DIE, distance-independent esotropia; ARDE, age-related distance esotropia; Δ, prism diopter (+: eso, -: exo)

**, *p*<0.01.

### Position of the extraocular muscles

**Fig 4:**
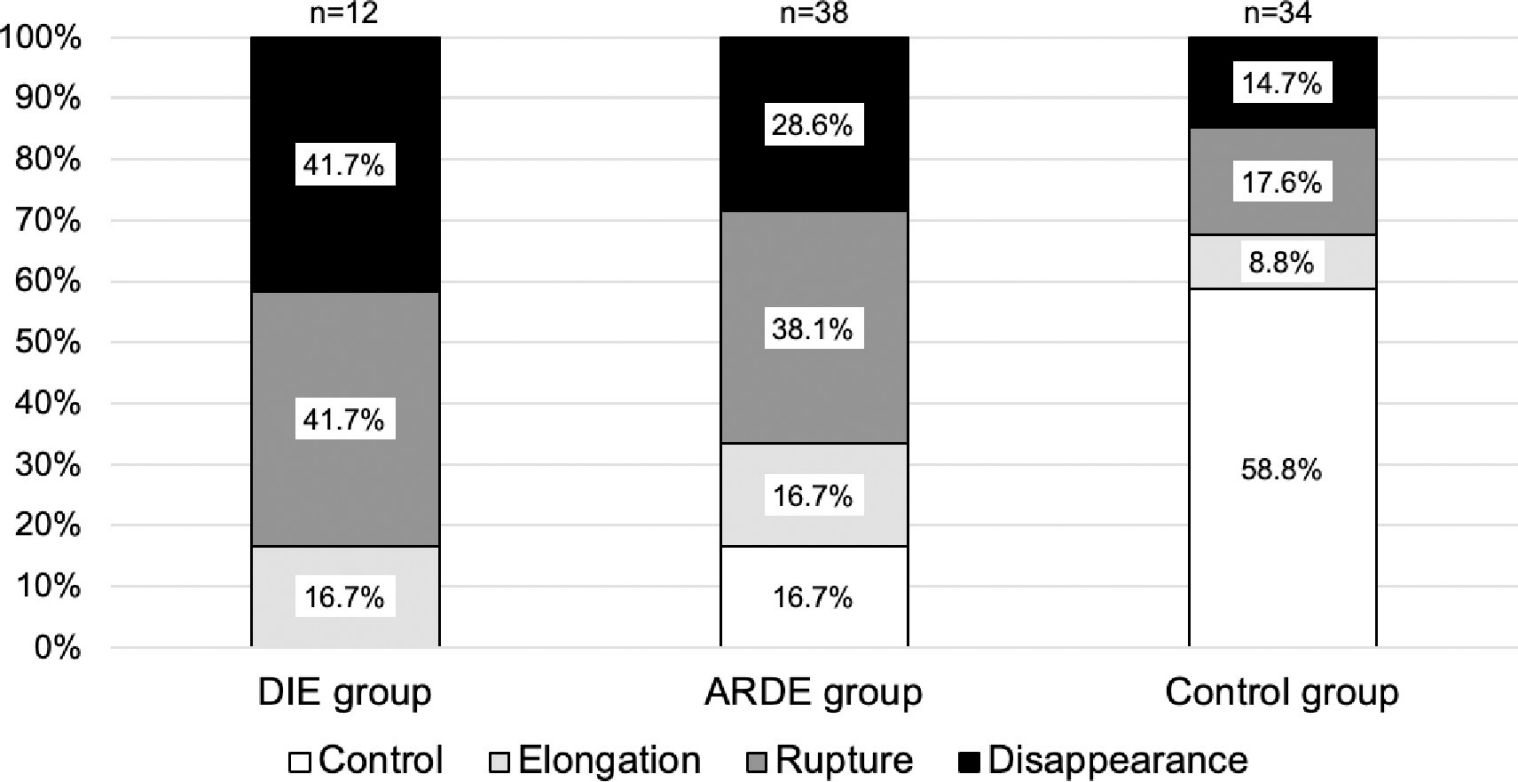
Condition of the lateral rectus–superior rectus band. DIE, distance-independent esotropia; ARDE, age-related distance esotropia.

The LR-SR bands of the DIE and ARDE groups had higher abnormality rates compared to the control group (*p* < 0.01). The abnormality rates for the DIE and ARDE groups were comparable (Fig 4). The LR vertical angle shown in Table 2 was deviated downwards in the control, ARDE, and DIE groups (*p* < 0.05). However, there was no difference between the three groups for MR, SR, and IR. LR tilting angle was tilted temporally in the control, ARDE, and DIE groups (*p* < 0.01). The SR-LR displacement angle in the DIE group had a greater angle of dislocation compared to those of the control and ARDE groups (*p* < 0.05).

**Table 2:** Position of the extraocular muscles using orbital magnetic resonance imaging in each group.

		DIE	ARDE	Control	*p* value		
					DIE vs ARDE	DIE vs Control	ARDE vs Control
**Vertical angle of horizontal muscle (°)**	**Medial**	-5.9±4.1	-5.6±5.3	-7.9±4.7	1.00	0.65	0.14
	**Lateral**	-18.8±5.7	-12.2±9.1	-7.5±5.1	0.02*	<0.01**	0.02*
**Horizontal angle of vertical muscle (°)**	**Superior**	+14.0±5.7	+12.1±6.5	+11.1±5.1	1.00	0.45	1.00
	**Inferior**	+19.4±4.7	+19.7±4.8	+17.3±7.2	1.00	0.87	0.27
**LR tilting angle (°)**		-28.6±5.4	-20.0±8.6	-12.2±9.1	<0.01**	<0.01**	<0.01**
**SR-LR displacement angle (°)**		122.2±6.5	113.2±12.8	108.3±7.2	0.02*	<0.01**	0.13

Vertical angle of horizontal muscle: +, superior; -, inferior.

Horizontal angle of vertical muscle, LR tilting angle: +, nasally; -, temporally.

DIE, distance-independent esotropia; ARDE, age-related distance esotropia; LR, lateral rectus; SR, superior rectus

*, *p*<0.05

**, *p*<0.01.

### Orbit measurements

The proptosis was lower in the DIE and ARDE groups compared to the control group (*p* < 0.01). The volume of the globe and the bony orbit cavity were not different between the three groups. However, the volume of the whole orbit was smaller in the DIE group than in the control group (*p* < 0.05). There was no difference in G/BO between the three groups; however, G/WO was higher in both DIE and ARDE groups than in the control group (*p* < 0.01) (Table 3).

**Table 3:** Proptosis and volume measurements using orbital magnetic resonance imaging in each group.

	DIE	ARDE	Control	*p* value		
				DIE vs ARDE	DIE vs Control	ARDE vs Control
**Proptosis (mm)**	11.4±2.6	12.5±2.3	14.7±2.6	0.50	<0.01**	<0.01**
**Volume of globe (mm**^**3**^**)**	8541.2±636.9	8992.0±1185.2	8627.9±889.1	0.54	1.00	0.39
**Volume of bony orbit cavity (mm**^**3**^**)**	24964.0±1514.2	26337.2±4302.7	25541.4±3802.0	0.85	1.00	1.00
**Volume of whole orbit (mm**^**3**^**)**	30933.2±2725.2	33867.9±5502.7	35270.5±5169.8	0.25	0.04*	0.74
**G/BO**	0.34±0.02	0.34±0.03	0.34±0.04	1.00	1.00	1.00
**G/WO**	0.28±0.01	0.27±0.02	0.25±0.03	0.69	<0.01**	<0.01**

DIE, distance-independent esotropia; ARDE, age-related distance esotropia; G/BO, globe/bony orbit cavity; G/WO, globe/whole orbit

*, *p*<0.05

**, *p*<0.01.

## Discussion

This is the first study to compare the orbital MRI characteristics of ARDE and acquired esotropia both at distance and near. The DIE group showed abnormal LR-SR bands, malpositions of the LR, and low proptosis and reduced intra-orbital volume in comparison with the ARDE and control groups. The underlying pathology of abnormal orbital connective tissue and displacement of extraocular muscle may be more severe in DIE than in ARDE.

Comparison of orbital MRI findings showed a higher rate of LR-SR band abnormalities in the ARDE and DIE groups than in the control group. The LR-SR band in patients with ARDE had previously shown a higher rate of abnormality than that in normal elderly patients [4,17]. Several previous reports of acquired esotropia with large deviation angles in the absence of high myopia showed clinical characteristics similar to that of the DIE group in this study. The orbital MRI findings in these reports suggested thinning and elongation [24] or disappearance [25] of the LR-SR bands. The present study was consistent with the finding that orbital connective tissue damage was one of the factors in DIE as well as ARDE.

Regarding the vertical deviation of the LR in the present study, the DIE group showed the greatest downward deviation, and the LR was tilted towards the temporal side. Additionally, there was no difference in the location of the other rectus muscles. In a previous study, we compared the position of the LR in ARDE with that in normal elderly participants using the same MRI measurement and analysis method as in the present study, and our results showed that the LR was significantly deviated downwards and tilted temporally in ARDE [17]. A case report of acquired esotropia with similar findings to DIE in this study showed malposition of the LR and SR [26], inferior deviation of the LR [25], and nasal deviation of the SR and IR [24,27]. A significant correlation was observed between the binocular difference in the horizontal rectus sag and the deviation angle in cyclo-vertical strabismus [4]. Therefore, the deviation angle and malposition of extraocular muscles were considered to be related in cases of DIE as well. In the present study, malposition of the LR was common, although there were differences at the unified measurement site 3 to 6 mm anterior to the optic nerve attachment, and larger deviation angles were observed in the previously reported cases.

A diagnosis of ARDE excludes other diseases, including abduction nerve palsy, indicating that there is no apparent abduction restriction and that the saccade is normal [28]. Meanwhile, HES is caused by the dislocation of the posterior part of the globe, resulting in mechanical eye movement disorders and varying degrees of abduction restriction [29]. All patients with DIE in the present study had abduction restrictions, similar to a report by Serafino et al. describing acquired esotropia without high myopia [26]. This study excluded patients with axial high myopia, which may have limited the mobility of eye movement due to the large degree of abnormalities in the LR position.

There was no difference in age or in the interval from the onset of diplopia to the visit to our hospital between the ARDE and DIE groups. In a previous report, the progress of the deviation angle in the ARDE group (5 years) was 3.4 Δ at distance and 2.7 Δ at near [30], with a small angle but progressing or increasing. Moreover, 16% of patients had recurrent diplopia after surgery for ARDE [31], and ARDE is expected to progress during observation. The findings of the present study and those of previous reports raise speculation over whether the reasons for the large deviation of the strabismus angle and malposition of extraocular muscle were due to connective tissue instability [32] or individual differences.

The ARDE and DIE groups in the present study showed less proptosis than the control group. Esotropic eyes of the DIE and ARDE groups will underestimate the measurement of the perpendicular distance from the top of the cornea to the line on axial magnetic resonance imaging. There was also no difference in G/BO, although G/WO was large; thus, the proportion of the whole orbit against the globe was small. SES presents with age-related external ocular adnexal findings such as superior sulcus deformity, aponeurotic ptosis, or high lid crease as facial features [3,4,33]. The lower proptosis and greater G/WO in the ARDE and DIE groups may be related to these facial features. Since the amount of connective tissue in the orbit decreases with age [34], we speculate that the DIE group had a decrease in whole orbital volume and also a concomitantly lower proptosis, similar to the pathogenesis of SES.

The findings of this study must be considered within the context of its limitations. First, the control group included patients with optic neuritis. Although an effect of optic nerve inflammation cannot be completely ruled out, we do not think it had any effect on the pulley, extraocular muscles, or ocular volume. Second, the MRI scans were taken using two MRI scans with different parameters. Additionally, since the MRI parameters have a slice thickness of 3 mm and slice gaps of 1 mm, the values of the volume are approximate. Third, since cases of DIE are rare, we did not calculate the sample size for this study. The number of cases in the DIE group was small in this study, and a larger number of cases are needed in future studies.

## Conclusion

Acquired esotropia both at distance and near presented with abnormalities of the LR-SR band, inferior deviation, and a tilt toward the temporal side of the LR, but the position of the other rectus muscles did not differ from those in the ARDE and control groups. The proptosis was low and the proportion of the whole orbit to the globe was small, similar to the facial features of SES. We consider that DIE is a severe form of ARDE. Thus, treatment and surgical technique selection for DIE should be based on these clinical findings.

## Supporting information

S1 File The data analyzed in the current study. (XLSX)
